# Complete characteristics and phylogenetic relationships of the *Garrulax albogularis* mitochondrial genome (Passeriformes: Timaliidae)

**DOI:** 10.1080/23802359.2018.1532834

**Published:** 2018-10-29

**Authors:** Xue Liu, Huailiang Xu, Yanyan Zhou, Diyan Li, Qingyong Ni, Mingwang Zhang, Meng Xie, Anxiang Wen, Qing Wang, Jiayun Wu, Yongfang Yao

**Affiliations:** a College of Life Science, Sichuan Agricultural University, Ya’an, China;; b College of Animal Science and Technology, Sichuan Agricultural University, Chengdu, China

**Keywords:** *Garrulax albogularis*, mitochondrial genome, phylogenetic analysis

## Abstract

In this study, we sequenced the complete mitochondrial genome of the bird *Garrulax albogularis.* The mitochondrial genome of *G. albogularis* was 17,870 bp and contained 13 protein-coding genes, 22 transfer RNA genes, two ribosomal RNA genes and two control regions. The overall base composition of the mitogenome was biased toward at 53.83%A + T content. ND6 and nine tRNA genes were encoded on the L-strand. The other protein-coding genes (PCGs) and tRNA genes were all distributed on the H-strand.

The white-throated laughingthrush (*Garrulax albogularis*) is a member of the Timaliidae family. With a white breast, brown nape and crown, *G. albogularis* is easily identified. The white-throated laughingthrush was mainly native to Bhutan, China, India, Nepal, Pakistan and Vietnam. Though it is classified as needing the least concern (LC) on the Red List by the International union for Conservation of Nature (IUCN), the distribution of *G. albogularis* is unbalanced. In India, they are considered very rare and while in Pakistan, the white-throated laughing thrush is possibly extinct. However, *G. albogularis* was widespread in Nepal, Bhutan and, locally common in China (del Hoyo et al. 2007). The differences between populations in different areas were not significant and its subspecies differentiation is still controversial (Zheng et al. 1987; del Hoyo et al. 2007). In our study, the complete mitochondrial genome was sequenced. Mitochondrial DNA sequencing could provide the genetic information for the phylogenetic analysis of *G. albogularis.*

The muscle sample of *G. albogularis* was collected from Ya’an, Sichuan province of China (N30anced E102ancedi The samples were then stored at the Wildlife Conservation Laboratory at Sichuan Agricultural University, Sichuan province, China. The whole genome DNA (gDNA) was extracted from the muscle tissue using phenol-chloroform. The complete mitochondrial genome sequence of *G. albogularis* was amplified and sequenced with 20 pairs of primers with normal PCR methods (GeneBank accession no. NC_037464). We constructed the neighbour-joining (NJ) tree by MAGE6.0.

The complete mitochondrial genome of *G. albogularis* was 17870 bp in total length. Similar to other Timaliidae mitogenomes (Zhang et al. 2014; Li et al. 2017), it contained 13 typical protein-coding genes (PCGs), 22 tRNA genes, two rRNA genes (12S rRNA and 16SrRNA) and two control region (D-loop1 and D-loop2). ND6 and nine tRNA genes were encoded on the L-strand. The other genes were encoded on the H-strand. The base composition of mtDNA was 30.13% A, 23.69% T, 32.29% C and 13.88% G. The percentage of A + T (53.83%) was slightly higher than G + C (46.17%). All PCGs began with ATG expect for the COX1 gene which started with GTG. Four PCGs (ND2, COX3, ND3 and ND4) showed incomplete stop codons (T or TA). Five genes - COX2, ATP8, ATP6, ND4 and Cytb – ended with TAA. ND1 and ND5 stopped with AGA. COX1 stopped with AGG. ND6 terminated with TGA, which was the same as *Pomatorhinus ruficollis* and *Garrulax elliotii* (Zhao et al. 2016; Zhou et al. 2016).

The two control regions of the *G. albogularis* mitochondrial genome were D-loop1 (1122 bp) and D-loop2 (1173bp). The 12S rRNA (980 bp) and 16S rRNA (1597 bp) genes were located between the tRNA*^phe^* and tRNA*^Leu^*^(^*^UUR^*^)^genes, which were separated by the tRNA*^Val^* gene.

In Figure 1, *G. albogularis* was closest to the other four birds in the *Garrulax* genus and to the Timaliinae and Passeriformes birds, respectively. Therefore, it is reasonable to divide *G. albogularis* between Timaliinae and the *Garrulax.*

**Figure 1.**
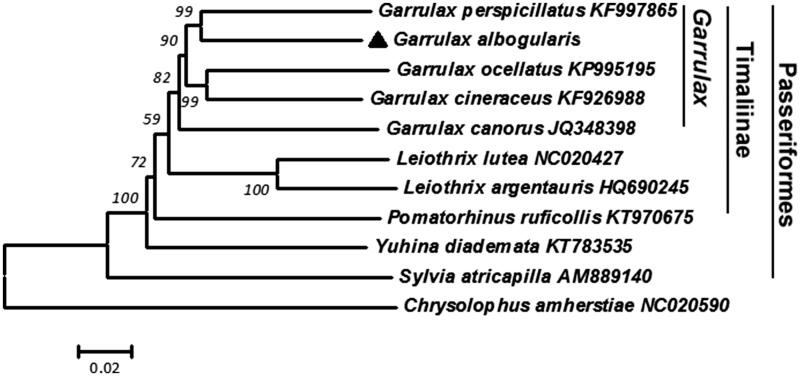
Neighbour-joining (NJ) tree based on combining 13 protein-coding gene sequences of 11 species by using MEGA 6.0 program. The NJ bootstrap for 10,000 replicates was indicated in each node. The *Chrysolophus amherstiae* was chosen as the outgroup.

## References

[CIT0002] del Hoyo J, Elliott A, Christie D 2007 Handbook of the birds of the world. Vol. 12: Picathartes to tits and chickadees. Barcelona, Spain: Lynx Edicions.

[CIT0003] Li B, Yao YF, Li DY, Ni QY, Zhang MW, Xie M, Xu HL 2017 The complete mitochondrial genome sequence of White-collared Yuhina (*Yuhina diademata*). DNA Sequence. 28:21–22. 10.3109/19401736.2015.1106524 26641536

[CIT0001] Zheng TH, Long ZY, Zheng BL 1987. Fauna Sinica (Aves, Vol 11 Passeriformes, Muscicapidae II Timaliinae). Beijing: Science press; p. 85–88.

[CIT0005] Zhang H, Li Y, Wu X, Xue H, Yan P, Wu X 2014 The complete mitochondrial genome of *Garrulax perspicillatus* (Passeriformes, Timaliidae). Mitochondrial DNA. 27:1. 25090386 10.3109/19401736.2014.945548

[CIT0006] Zhao Q, Xu HL, Li B, Xie M, Li DY, Ni QY, Zhang MW, Yao YF 2016 Characterization of the complete mitochondrial genome and phylogenetic relationship of *Pomatorhinus ruficollis* (Passeriformes, Timaliinae). Mitochondrial DNA. 1:150–151. 10.1080/23802359.2016.1144100 PMC7800912 33473438

[CIT0007] Zhou YY, Wei DJ, Qi Y, Xu HL, Li DY, Ni QY, Zhang MW, Yao YF 2016 Complete mitochondrial genome of G*arrulax elliotii* (Passeriformes, Timaliidae). Mitochondrial DNA Part A: DNA Map Sequencing Anal. 27:3687. 10.3109/19401736.2015.1079856 26366960

